# Colonization by Chironomidae Larvae in Decomposition Leaves of *Eichhornia azurea* in a Lentic System in Southeastern Brazil

**DOI:** 10.1673/031.013.2001

**Published:** 2013-03-16

**Authors:** Lidimara Souza da Silveira, Renato Tavares Martins, Guilherme Augusto da Silveira, Richard Michael Grazul, Danielle Pinheiro Lobo, Roberto da Gama Alves

**Affiliations:** 1 Laboratóries de Invertebrados Bentônicos, Programa de Pós-graduação em Ciências Biológicas - Comportamento e Biologia Animal, Departamento de Zoologia, Instituto de Ciências Biológicas, Universidade Federal de Juiz de Fora. 36036-330 Juiz de Fora, Minas Gerais, Brasil; 2 Departamento de Química, Instituto de Ciências Exatas, Universidade Federal de Juiz de Fora. 36036-330 Juiz de Fora, Minas Gerais, Brasil

**Keywords:** aquatic insects, lake, macrophytes, tropical region

## Abstract

The objective of this study was to analyze the colonization of Chironomidae (Diptera) larvae during the decomposition of *Eichhornia azurea* (Swartz) Kunth (Commelinales: Pontederiaceae) leaves in a lake in southeastern Brazil in two seasons of the year. The experiment was conducted from September to November 2007 and February to April 2008. In each period, 21 litter bags were used, each containing 10 g of dried leaves. Three bags were removed after 2, 5, 8, 12, 25, 45, and 65 days of colonization. The decomposition rate of the *E. azurea* leaves was rapid in both seasons, with no significant difference between them. The Chironomidae showed higher density than the other invertebrates. *Goeldichironomus, Tonytarsus*, and *Corynoneura* were the most abundant genera of Chironomidae. The invertebrate density increased during the experiment, differing within days but not between seasons. The faunal composition differed between the decomposition phases (initial and final), but did not differ between the seasons (dry and wet). The taxa *Ablabesmyia, Caladomyia, Chironomus, Goeldichironomus*, and *Parachironomus* were the most closely related to the final days of the experiment. Litter was the main food item found in the gut contents of the organisms of all the genera analyzed, both at the beginning and end of the decomposition. We believe that the feeding activity combined with the high larval density is an important factor contributing to the rapid decomposition of the *E. azurea* leaves. In conclusion, the succession process along the detritus chain of *E. azurea* was more important in structuring the assemblage of Chironomidae larvae than seasonal variations.

## Introduction

The macrophyte *Eichhornia azurea* (Swartz) Kunth (Commelinales: Pontederiaceae) is widely distributed in Neotropical regions, including throughout Brazil (Barret 1978; Alves dos Santos 1999). Aquatic plants play a fundamental role in various ecological processes, with the decomposition of their biomass being one of the main routes for cycling organic matter (Azevedo et al. 2008). Additionally, macrophytes are important sources of litter (Pieczyfiska 1986), utilized as food and shelter by aquatic invertebrates (Gonçalves et al. 2000).

The decomposition process depends on many factors, but invertebrates are a key component of this process, playing important roles such as leaf litter fragmentation, which increases the area available for decomposing microorganism's colonization, and increasing the nutritional content of the litter by depositing their excreta on the leaf fragments (Graça 2001). Moreover, invertebrates contribute greatly to leaf mass loss, even when compared to powerful decomposers such as fungi and bacteria (Hieber and Gessner 2002). However, the role of shredding invertebrates, especially in tropical regions, is still not very clear (Mathuriau and Chauvet 2002; Gonçalves et al. 2006).

Chironomidae (Diptera) are often the main invertebrates found colonizing macrophytes in decomposition experiments (Nessimian and De Lima 1997; Gonçalves et al. 2004). Larvae of this family have highly diverse feeding habits (collector-gatherers, collector-filterers, scrapers, shredders, engulfers, and piercers) and ingest a wide variety of foods (algae, detritus, macrophytes, woody debris, and animal matter) (Berg 1995). Callisto et al. (2007) showed that some Chironomidae species are able to use leaves as a food source and concluded that the participation of these larvae in the decomposition of leaves depends on their density, the leaf quality, and the presence of other consumers that use large organic particles as a food source. According to Galizzi and Marchese (2007), some Chironomidae taxa (*Polypedilum* spp., *Phaenopsectra*, and *Endotribelos* spp.) act as shredders, accelerating the decomposition process, since they are observed in high abundance forming tunnels in the mesophyll tissue of leaves.

The limnological characteristics of the water, such as the concentrations of dissolved oxygen and nutrients as well as temperature and pH, influence both the decomposition process (Webster and Benfield 1986; Magee 1993; Cunha-Santino and Bianchini 2006) and the makeup of the aquatic community. The alteration of these variables occurs seasonally, so there should be differences in the decomposition rate and faunal composition between seasons of the year. Brock et al. (1985) observed greater weight loss of leaves in hotter periods, showing the effect of temperature on the leaf decomposition process. Temperature mainly affects the activity of microorganisms, while feeding by invertebrates appears to be less influenced by temperature (Webster and Benfield 1986).

The association between the aquatic invertebrate community and the plant parts during the decomposition process has been widely studied. However, few studies have assessed this association in different seasons of the year. In Brazil, such studies were conducted by Stripari and Henry (2002) and Rezende et al. (2010) in different tropical lentic systems.

The objective of our study was to analyze the colonization of Chironomidae larvae during the decomposition of *E. azurea* leaves in the dry and wet seasons. In the work of Gonçalves et al. (2004), the main driving force structuring the invertebrate's community was degradative ecological succession, where the improvement in the nutritional quality of the substrate during the decomposition process allows the establishment of a greater number of individuals and invertebrate taxa. Thus, we expected an increase in the density and richness of Chironomidae during the degradation of leaves of *E. azurea.* Additionally, we expected higher rates of decomposition and invertebrate colonization during the summer, due to the increase in temperature and activity of microorganisms in the debris.

## Materials and Methods

### Study area

**Figure 1.**
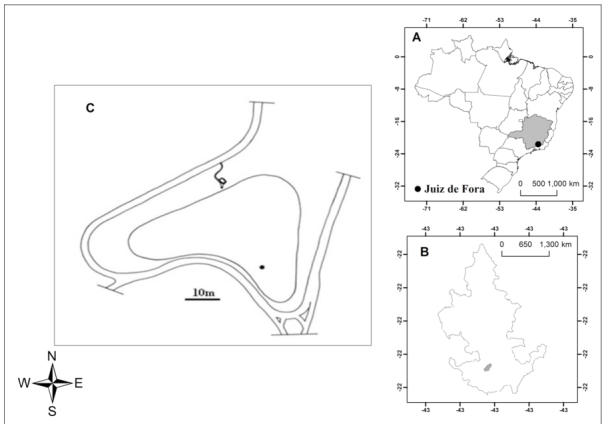
A- Brazil, with Minas Gerais in gray and highlighted Juiz de Fora, B- Juiz de Fora and highlighted the Universidade Federal de Juiz de Fora, C- Manacas Lake, the study area (Azevedo et al. 2003). High quality figures are available online.

The experiment was conducted in Manacás Lake (21° 46′ 68″ S, 43° 22′ 22″ W), a reservoir with a surface area of 0.02 km^2^ and a maximum depth of 5 m (Azevedo et al. 2003), located in the municipality of Juiz de Fora, Minas Gerais state, in southeastern Brazil (Figure 1).

The information described below was obtained from Martins et al. (2011). The lake's water is turbid (Secchi disk: 0.60 ± 0.12 m), with neutral pH (7.10 ± 0.25) and average levels of dissolved oxygen, temperature and electrical conductivity of 5.55 ± 2.04 mg.L^-1^, 21.35 ± 2.25°C and 28.25 ± 12.82 µS.com^-1^, respectively. Blooms of *Salvinia* spp. generally occur in this lake in the summer. However, we did not observe these macrophytes during the experiment. The terrestrial vegetation in the immediately surrounding area consists mainly of *Merostachys* sp. Sprengel (Poales: Poaceae) and *Tibouchina granulose* Cogniaux (Myrtales: Melastomataceae). Soares et al. (2009), in another study in Manacás Lake, verified an increase in algal biomass, mainly cyanobacteria, in the winter as a function of the seasonal turnover of water levels and the greater availability of nutrients.

### Collection and analysis of the data

Leaves of *E. azurea* were collected from a nearby lake (21° 52′ 49.6″ S, 43° 00′ 28.71″ W) and washed to remove the adhered material. The leaves were air-dried and then placed in an oven at 60° C for 48 hrs to obtain dry mass before being immersed in the lake (Ramseyer and Marchese 2009). The experiment was conducted from September through November 2007 (dry season) and February through April 2008 (wet season). In each period, 21 litter bags (15 × 15 cm and 2 mm mesh) were used, each filled with 10g of leaves and placed near the sediment. Three bags were removed 2, 5, 8, 12, 25, 45, and 65 days after immersion in the lake.

The remaining material in each bag was fixed in 4% formol and passed through a 0.21 mm sieve. The remaining plant material was dried at 60° C in an oven for 48 hrs, and the breakdown rate of the macrophyte was calculated using a negative exponential equation (e.g., Petersen and Cummins 1974). The invertebrates were separated under a stereoscopic microscope and preserved in 70% alcohol for subsequent identification. The identification was carried out to the genus level according to the taxonomic criteria proposed by Wilderholm (1983), Epler (1992), and Trivinho-Strixino and Strixino (1995). The gut contents of the some Chironomidae larvae were examined under a microscope (1000x) by transparency through the cuticle. The percentage composition of each food item was tabulated by genus and decomposition phase (initial and final). The fauna structure was verified by calculating the mean numerical density and the taxonomic richness (number of Chironomidae taxa) for each day and season.

The Mann-Whitney test was used to verify whether the air temperature and precipitation differed between the seasons. Analysis of Covariance (ANCOVA; Zar 2010) was used for comparing the lost mass and the invertebrate density (dependent variables) between seasons (categorical variable), with days used as the covariate. These analyses were performed with the STATISTICA version 7 program (Statsoft Inc. 2004).

Detrended Correspondence Analysis (DCA; Hill and Gauch 1980) was used to order the days and seasons of the colonization experiment, seeking to group the most similar ones in relation to the composition and density of Chironomidae larvae. This analysis was performed with the PC-ORD version 5.15 program (McCune and Mefford 2006). The Analysis of Similarity (ANOSIM; Clarke 1993) was performed to verify that there is significant difference in the composition of Chironomidae between days groups formed in DCA and between the stations in the program R (R Foundation For Statistical Computing 2011). The statistical value R varies between 1 and 1. When samples of one group are completely different from samples of a different group, the distances “between groups” will always be greater and R is 1. If the groups do not differ, the distances “between groups” will be similar to the distances “within groups” and the value of R is close to 0. Negative values can be obtained, but have no simple interpretation (Melo and Hepp 2008).

Indicator Species Analysis (Dufrêne and Legendre 1997) was used to verify which taxa were more important to the Chironomidae community structure. This method combines the relative abundance and frequency of occurrence of each taxon in different sample groups. These groups were established *a priori* based on the groups formed in the DCA. An indicator value is calculated for each species in each group and these are tested for statistical significance using a randomization technique. This analysis was performed in the PC-ORD version 5.15 program (McCune and Mefford 2006).

## Results

### Air temperature and precipitation

During the dry season, the average temperature and precipitation were 20.92 ± 2.80^°^C and 104.13 ± 89.41 mm respectively, while during the wet season these values were 21.85 ± 1.94^°^C and 254.90 ± 118.96 mm respectively. There were significant differences in precipitation (Z = 3.11; *p* < 0.01) and temperature (Z = 2.82; *p* < 0.01) between the two seasons.

### Decomposition

**Figure 2.**
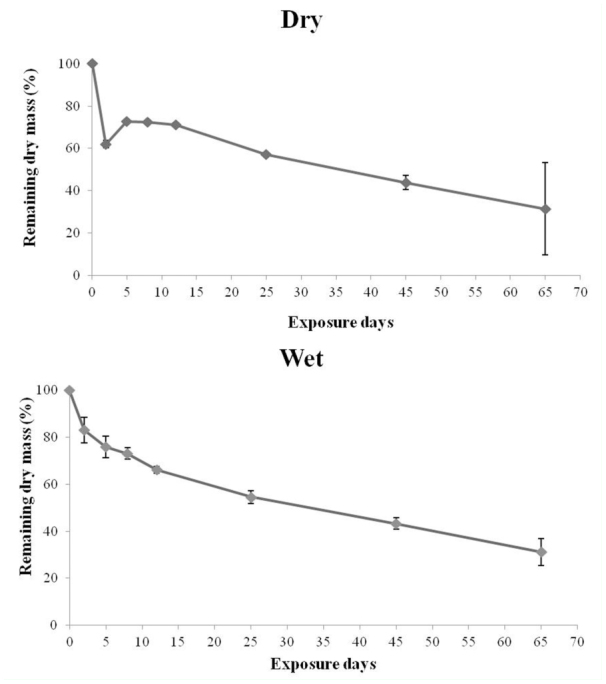
Remaining dry mass (%) during the colonization experiment with *Eichhornia azurea* leaves in Manacás Lake (southeastern Brazil). High quality figures are available online.

In the first two days, there was rapid mass loss both in the dry (37.97%) and wet (17.00%) season, with a significant difference in mass loss between days (ANCOVA, F_1,41_ = 202.83; *p* < 0.01). At the end of the experiment, the remaining mass values were 3.14 g DM (31.40%, dry season) and 3.11 g DM (31.10%, wet season) (Figure 2). There was no difference in mass loss between stations (ANCOVA, F_1,41_ = 3.16; *p* = 0.08), and the decomposition rate was 0.023 d^-1^ in the dry season and 0.018 d^-1^ in the wet season. The time necessary for the decomposition of 50% of the initial biomass was 30 and 39 days for the dry and wet seasons respectively. The corresponding time for 95% decomposition was 131 and 167 days for the dry and wet seasons respectively.

### Chironomidae

**Figure 3.**
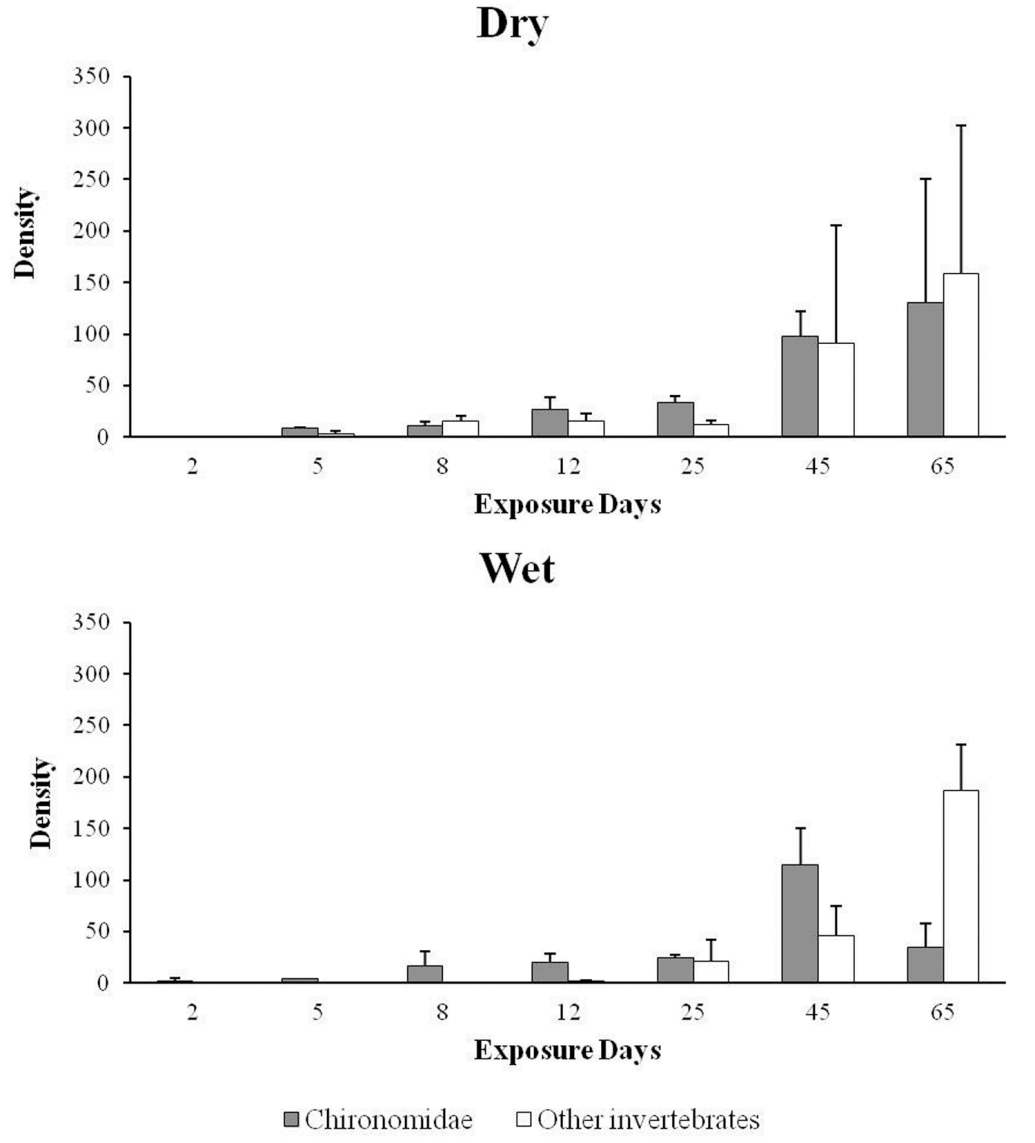
Densities (average of three repetitions ± standard deviation, ind.g^-1^ DM) of Chironomidae and other invertebrates during the decomposition experiment with *Eichhornia azurea* leaves in Manacás Lake (southeastern Brazil). High quality figures are available online.

During the experiment, we identified 12,472 invertebrates, of them 6,689 were Chironomidae and 5,783 were other invertebrates. Besides the Chironomidae, in the dry season we found 3,015 invertebrates, distributed among Diptera (54.19%), Oligochaeta (45.58%), Trichoptera (0.20%), and Coleoptera (0.03%). The corresponding numbers in the wet season were 2,768 invertebrates, distributed among Diptera (53.11%), Oligochaeta (46.41%), Trichoptera (0.37%), Odonata (0.05%), and Ephemeropetra (0.05%). The Chironomidae showed higher density than the other invertebrates, except for Days 8 and 65 of the dry season and Day 65 of the wet season (Figure 3).

**Figure 4.**
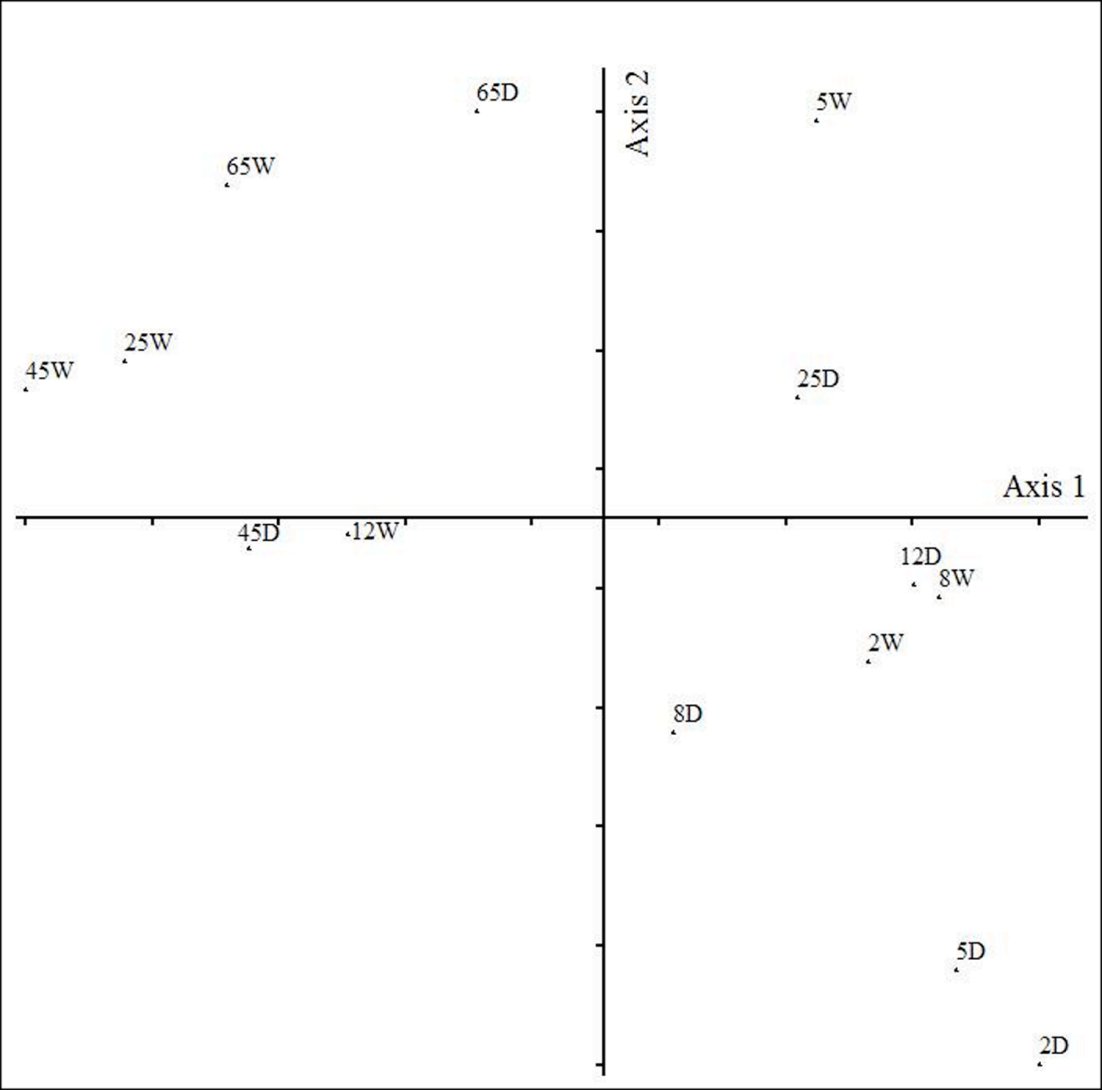
Detrended correspondence analysis of the days in the dry (D) and wet (W) season during the colonization experiment with *Eicchornia azurea* leaves in Manacás Lake (southeastern Brazil). High quality figures are available online.

**Table 1.**
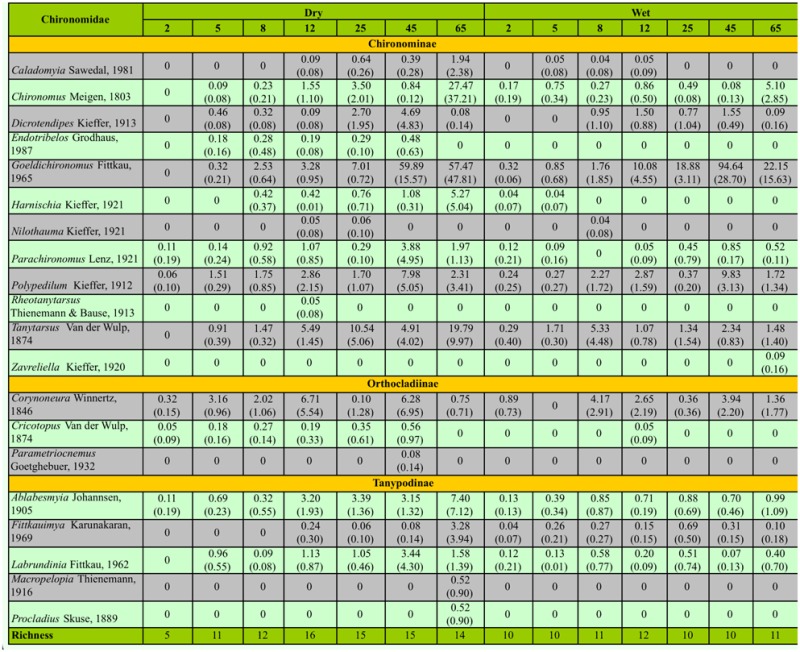
Taxonomic composition, density (mean of three repetitions ± standard deviation, ind.g^-1^ DM), and taxonomic richness of Chironomidae larvae during the colonization experiment with *Eicchornia azurea* leaves in Manacás Lake (southeastern Brazil).

A total of 3,558 Chironomidae larvae were found in the dry season and 3,131 in the wet season, distributed in 20 genera (dry = 19; wet =15; Table 1). The most abundant genera were *Goeldichironomus* (dry = 38.65%; wet = 63.94%), *Tanytarsus* (dry = 15.65%; wet = 8.02%), and *Corynoneura* (dry = 10.40%; wet = 7.51%). The densities of the Chironomidae were 28.86 ind.g^-1^ DM (dry season) and 24.44 ind.g^-1^ DM (wet season). The invertebrate density increased during the experiment, showing difference among the days (ANCOVA, F_1,41_ = 35.53; *p* < 0.01) but not between seasons (ANCOVA, F_1,41_ = 0.53; *p* = 0.47).

The dispersion of the samples in two dimensions of space, according to the DCA, is presented in Figure 4. The first axis (eigen-value = 0.25) separated the samples on different days of the colonization experiment, but not between the collection seasons. The samples were separated into two groups: Days 2, 5, 8, and 12 presented the highest scores while those on Days 25, 45, and 65 had the lowest scores. The fauna composition was different between the two groups formed in the DCA (ANOSFM, R = 0.51; *p* < 0.01), but was not different between seasons (ANOSFM, R = -0.03; *p* = 0.55).

**Table 2.**
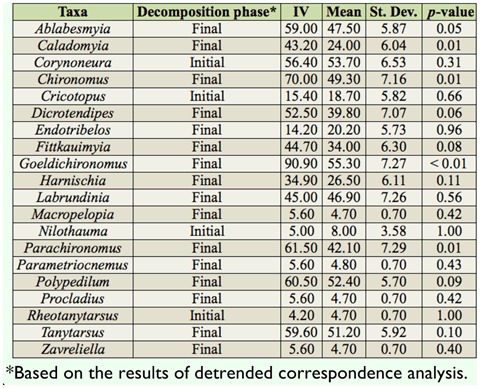
Indicator species analysis for the Chironomidae fauna collected during the colonization experiment with *Eicchornia azurea* leaves in Manacás Lake (southeastern Brazil).

The indicator species analysis did not point to the existence of any indicator genus during the initial days (2, 5, 8, and 12) of decomposition. However, five taxa (*Ablabesmyia, Caladomyia, Chironomus, Goeldichironomus*, and *Parachironomus*) were recorded in high abundance and frequency on the final days (25, 45, and 65) of the colonization experiment (Table 2).

The Chironomidae larvae were found to have ingested a variety of food items, but their diet mainly consisted of plant detritus at the beginning and end of the experiment. *Chironomus, Polypedilum*, and *Tanytarsus* showed more than 50% of debris in the digestive tract in the two phases of decomposition.

**Table 3.**
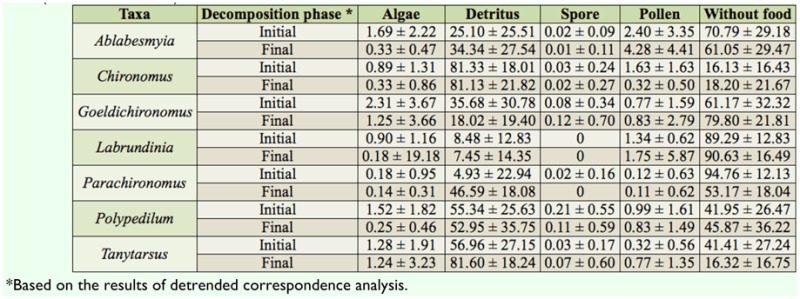
Food items (%) of Chironomidae genera during the colonizaton experiment with *Eicchornia azurea* leaves in Manacás Lake (southeastern Brazil).

In contrast, larvae of *Ablabesmyia, Goeldichironomus, Labrundinia*, and *Parachironomus* contained little food content in their digestive tract (Table 3).

## Discussion

### Decomposition

The decomposition of the *E. azurea* leaves was rapid in both seasons, according to the classification of Petersen and Cummins (1974). The loss of 37.97% (dry season) and 17.00% (wet season) of initial mass of the leaves in the first two days may be related to the method used to dry the material (in an oven at 60°C) before immersion in the lake, possibly causing rupture of the plant cells (Gessner et al. 1999). Afterward, the decomposition became slower, which according to Nessimian and De Lima (1997) happens due to the presence of a relatively larger quantity of structural compounds, such as cellulose and lignin.

The similar decomposition rate and time necessary for decomposition of 50% and 95% of the biomass of the macrophyte studied in the two seasons probably occurred due to the fact the experiment was conducted in a reservoir with controlled water release. Therefore, the difference in precipitation between the two seasons did not greatly influence the decomposition process since the water level in the lake did not change. Although physical abrasion apparently has little effect on the decomposition of organic matter in lakes, Pabst et al. (2008) found that waves and water circulation can have a substantial effect on the processing of detritus in these systems. Stripari and Henry (2002) studied the decomposition of *E. azurea* in a lake in the state of São Paulo and also verified no difference in the decomposition rate between the dry (K = 0.0135 d^-1^) and wet (K = 0.0175 d^-1^) seasons, even with higher water temperatures in the wet season. Thus, the decomposition process in lakes seems to not be affected by seasonal difference.

### Chironomidae

The Chironomidae family occurred in greater density than the other invertebrates on the majority of days in the two seasons. Larvae of this family disperse efficiently, through oviposition and/or the ability to swim (Oliver 1971), enabling them to colonize the substrate rapidly (Poi de Neiff and Bruquetas de Zozaya 1991). The proximity of the litter bags to the lake sediment might have facilitated their colonization. Additionally, litter bags function as filtering nets, accumulating detritus from the environment and released by the plant material (Mormul et al. 2006). This fact, combined with the low abundance of predator insects (Odonata) observed and the absence of macro-consumers (such as fish and shrimp) due to the mesh size of the litter bags, which does not allow animals to enter that are larger than 2 mm, might have contributed to the high density of the larvae of this family.

The taxa richness (19 taxa in the dry season and 15 in the wet season) was similar between seasons, showing the assembly of Chironomidae in a lentic environment is not influenced by seasonality, as lotic environments are, at least in the lake studied where no change occured in water level. Greater taxa richness is expected on intermediate days of such experiments, due to the greater spatial heterogeneity (Capello et al. 2004). The findings from this study corroborate with Capello et al. (2004), with the greatest taxa richness occurring on Day 12 in both seasons. Although we have not analyzed the microorganisms present in the debris of *E. azurea* and the quantity of nutrients, it is well established in the literature that decomposing leaves become a better nutritional resource for invertebrates as the degradation process advances as a consequence of the activity of bacteria and fungi (Smock and Stoneburner 1980), which increase the quantity of nitrogen and proteins during the decomposition (Suren and Lake 1989). Thus, the observed increase in density in the experiment, with significant variation between days verified by ANCOVA, probably occurred because of an improvement in the quality of the debris of *E. azurea.* Some authors have found an inverse relation between the remaining dry weight and density of invertebrates (Poi de Neiff and Bruquetas de Zozaya 1991; Stripari and Henry 2002; Silva et al. 2010), which emphasizes the greater importance of detritus to the fauna at the end of the decomposition process.

Changes in the composition and structure of the Chironomidae assemblage during the decomposition process of the *E. azurea* leaves were verified by the separation of the initial and final days of the experiment according to DCA and confirmed by ANOSIM. Differences between the days of the experiments occured because of changes in the structure, the consistency of the leaves, and the size of the detritus particles. Additionally, chemical alterations (Esteves and Barbieri 1983) can lead to difference in the community. According to Gonçalves et al. (2004), the relationship between substrate and invertebrates is very dynamic during the decomposition process, with the community of invertebrates being mainly structured by this process. ANOSIM did not show a difference in the faunal composition between the two seasons, probably due to the lack of difference in decomposition rate of this plant, and consequently in the food availability. Rezende et al. (2010) observed the highest decomposition rates and density of the invertebrate community in the wet season. According to the authors, this difference occurred because of the increase of temperature and the greater entry of nutrients and organic matter brought by runoff during the wet season.

As observed in the indicator species analysis, the taxa *Ablabesmyia, Caladomyia, Chironomus, Goeldichironomus*, and *Parachironomus* were more closely related in the final days of the experiment. These genera are common in the sediment (Trivinho-Strixino 2011), and were likely found in the litter bags due to their proximity to the substrate and the greater quantity and/or quality of the food in relation to the substrate. Some of these taxa are considered to be burrowers (*Goeldichironomus*) and detritivores (*Chironomus*), living in association with periphyton or detritus from macrophytes. These conditions most likely favored the increased density and frequency of these taxa at the end of the experiment. According to Capello et al. (2004), the activity of burrowing invertebrates is very important to accelerate the decomposition process. *Ablabesmyia* and *Parachironomus*, considered predators (Merrit and Cummins 1984), may have occurred in higher frequency and abundance in samples from the end of the experiment due to the increase in density of larvae.

The gut contents of the Chironomidae genera analyzed showed the presence of different food items (algae, detritus, spores, and pollen), but detritus was predominant, even for the genera considered to be predatory (*Ablabesmyia* and *Parachironomus*). The same result was obtained by Henriques-Oliveira et al. (2003), Sanseverino and Nessimian (2008), and Silva et al (2008). According to Berg (1995), few Chironomidae species are nutritionally selective. Rather, the great majority are generalists and opportunists. Therefore, irrespective of the stage of leaf decomposition, litter is an important food source for Chironomidae larvae, but leaves in an advanced decomposition stage most likely permit greater larval density.

We believe that the feeding activity and movement of the Chironomidae larvae, allied with their high density, are important factors to accelerate the decomposition of *E. azurea* leaves. Besides this, the results of the present study allow concluding that the degradation process was more important in structuring the Chironomidae assemblage than were seasonal variations (dry and wet). Nevertheless, further studies are needed, involving abiotic variables and different leaf constituents, to allow more comprehensive conclusions.
